# Chiral Aminophosphines as Catalysts for Enantioselective Double-Michael Indoline Syntheses

**DOI:** 10.3390/molecules17055626

**Published:** 2012-05-11

**Authors:** San N. Khong, Ohyun Kwon

**Affiliations:** Department of Chemistry and Biochemistry, University of California, Los Angeles, CA 90095, USA

**Keywords:** double-Michael reaction, chiral aminophosphines, anchimeric assistance, indoline

## Abstract

The bisphosphine-catalyzed double-Michael addition of dinucleophiles to electron-deficient acetylenes is an efficient process for the synthesis of many nitrogen-containing heterocycles. Because the resulting heterocycles contain at least one stereogenic center, this double-Michael reaction would be even more useful if an asymmetric variant of the reaction were to be developed. Aminophosphines can also facilitate the double-Michael reaction and chiral amines are more readily available in Nature and synthetically; therefore, in this study we prepared several new chiral aminophosphines. When employed in the asymmetric double-Michael reaction between *ortho*-tosylamidophenyl malonate and 3-butyn-2-one, the chiral aminophosphines produced indolines in excellent yields with moderate asymmetric induction.

## 1. Introduction

Most chiral natural products exist in enantiomerically pure forms, synthesized in Nature mostly through enzyme-mediated catalysis [1]. Not surprisingly, enantioselective catalysis processes were long believed, up until the early 1900s, to be accessible using only natural enzymes, until Bredig’s attempts at (i) kinetic resolution of racemic camphorcarboxylic acid through selective decarboxylation in a chiral medium (e.g., L- or D-limonene) or in the presence of a chiral alkaloid catalyst (e.g., nicotine or quinidine) [2,3,4,5] and (ii) asymmetric synthesis of mandelonitrile through the addition of HCN to benzaldehyde in the presence of a cinchona alkaloid catalyst (e.g., quinidine or quinine) [2,3,6]. Although these effortful studies were conceptually groundbreaking, the enantioselectivity achieved was synthetically impractical (<10% *ee*). A synthetically useful enantiomeric excess of 74% was first achieved by Pracejus in the synthesis of (–)-methyl α-phenylpropionate from methyl phenyl ketene and methanol with *O*-acetylquinine as the catalyst [7]. Catalytic asymmetric synthesis was established as a reliable and practical approach when Knowles reported, in the late 1960s and early 1970s, the first asymmetric hydrogenations catalyzed by transition metals bearing chiral phosphine ligands [8,9,10,11,12,13]. Shortly afterward, a great number of chiral phosphine ligands were developed to provide excellent enantioselectivity in transition metal-catalyzed hydrogenations [14,15]. The major disadvantage of transition metal catalysis, however, is that traces of toxic heavy metals can be left behind in the final products. 

In the early 1970s, L-proline-mediated asymmetric Robinson annulation was first reported [16,17,18,19], although the synthesis community paid very little attention to it because the reaction was considered a novelty. Since the concept of organocatalysis—the utilization of metal-free small organic molecules as catalysts for organic transformations—was developed and recognized in the late 1990s, there has been an explosion of research in the field of organocatalysis [20,21,22], which is now widely considered as a third main branch of research in asymmetric synthesis, beside enzymatic catalysis and organometallic catalysis.

**Figure 1:**
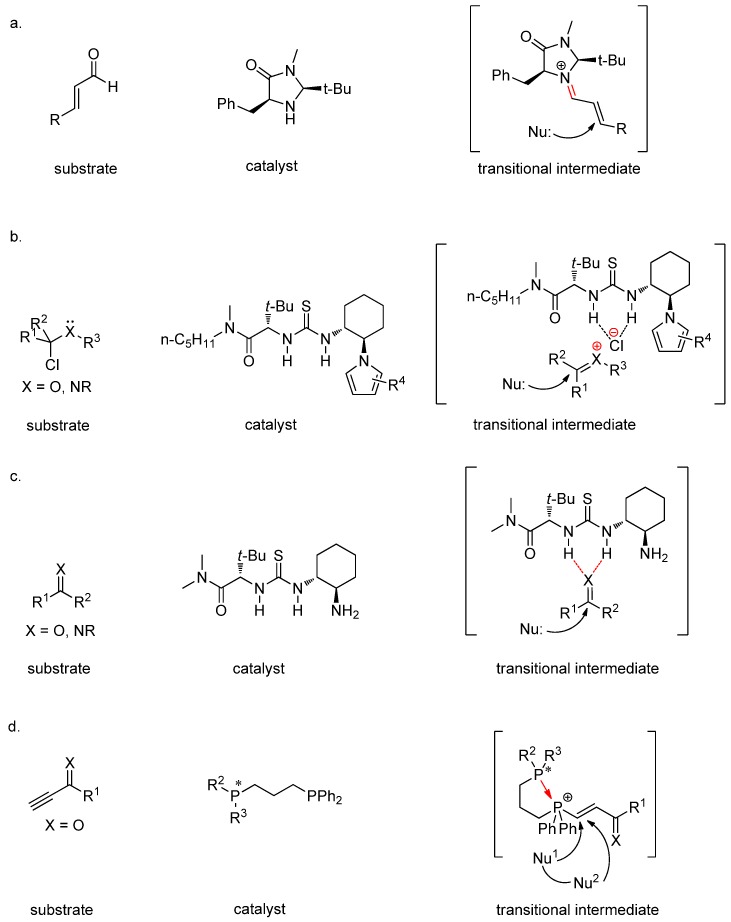
Modes of enantioselective induction provided by organocatalysts through (**a**) covalent bonding; (**b**) ionic bonding; (**c**) hydrogen bonding; and (**d**) intramolecular anchimeric assistance.

A chiral organocatalyst can render enantioinduction (asymmetric induction) through a few modes, including covalent bonding, ionic bonding, and hydrogen bonding (Figure 1a–c) [21]. In 2007, we reported a phosphine-catalyzed double-Michael addition in which a bisphosphine was the best catalyst [23,24,25]. We suggested that anchimeric assistance by the other tethered phosphino group stabilized the resultant phosphonium cation in the transitional intermediates, leading to a higher yield of the double-Michael adduct. Because of the rigid architecture provided by intramolecular anchimeric assistance, we suspected that a chiral element at the terminus of the non-reactive phosphino group would be likely to endow the reactive phosphonium center with steric bias, potentially leading to a new mode of enantioinduction (Figure 1d). Practically, the synthesis of chiral bisphosphines is often challenging because it requires careful handling of pyrophoric and air-sensitive phosphorus-containing intermediates.

**Figure 2:**
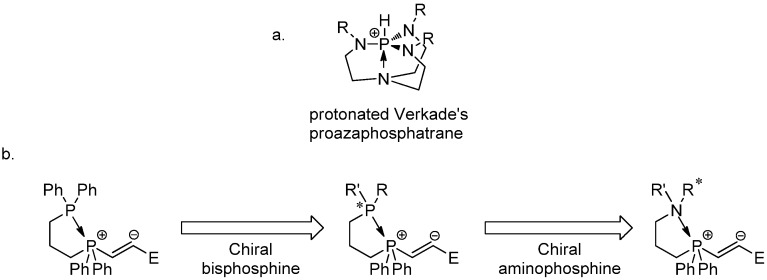
Possible intramolecular anchimeric assistance. (**a**) Verkade’s protonated proazaphosphatrane; (**b**) Utilization of a chiral aminophosphine instead of a chiral bisphosphine.

As a variant of the bisphosphine catalyst, we sought an equivalent to a phosphino group that would be easy to handle yet stabilize the tethered phosphonium cation through anchimeric assistance. In Verkade’s proazaphosphatrane [26,27,28], the nitrogen atom could efficiently stabilize the resultant protonated phosphonium center, making it a super base (Figure 2a). Therefore, we expected an aminophosphine—with an amino group replacing a tethered phosphino group in the bisphosphine—To function much like a bisphosphine when catalyzing double-Michael additions (Figure 2b). Less-toxic and more air-stable than phosphines, amines also have advantages in terms of their synthesis and storage. Furthermore, many diverse chiral amines are readily available commercially from natural sources. Appending a customized chiral element to an achiral amine is also easy to achieve through simple alkylation, imine reduction, or peptide coupling/reduction. Therefore, we proceeded to synthesize a variety of chiral aminophosphines to determine if they could result in enantioinduction via anchimeric assistance and concurrently to develop an asymmetric variant of the double-Michael reaction.

**Scheme 1:**
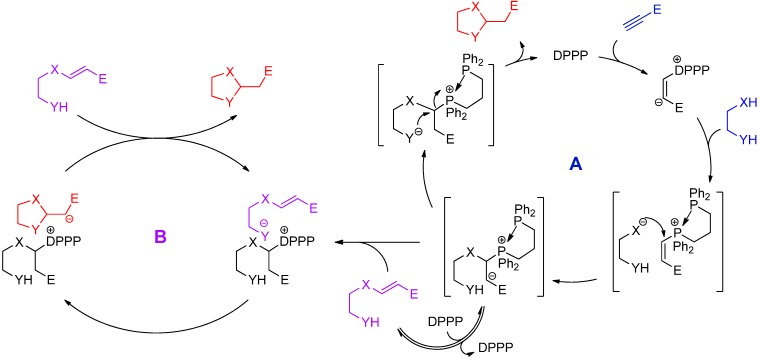
Possible mechanistic pathways for the double-Michael reaction.

We propose a reaction mechanism for the double-Michael reaction that occurs via pathway **A**, in which the cyclization is accomplished through direct intramolecular S_N_2 displacement of the phosphonium cation (Scheme 1). Alternatively, the reaction could possibly follow mechanistic pathway **B**, in which the intermediate phosphonium zwitterions serve as a Brønsted base and the resulting phosphonium species as a counter cation of the reactive intermediate anions (Scheme 1). In either case, the most important factor affecting the success of cyclization would be the stability of the phosphonium species generated along the pathway [23]. If the reaction employed a chiral aminophosphine catalyst, the enantioselectivity would probably be induced via anchimeric assistance of the amine in stabilizing the quaternary phosphonium center.

## 2. Results and Discussion

Chiral bisphosphines featuring *C*_2_- or local *C*_2_-symmetry, obtained directly from commercial sources, were tested in the double-Michael reaction between *o*-(*p*-tosylamido)phenylmalonate (**1a**) and 3-butyn-2-one (**2a**) to form the indoline product **3aa** (Table 1) [29]. The optimal conditions for the reaction catalyzed by diphenylphosphinopropane (DPPP) required an elevated temperature (entry 1). Employing chiral bisphosphines as catalysts, the double-Michael reaction at elevated temperature exhibited no enantioinduction (entries 2, 4, and 6). At room temperature, however, the reactions exhibited low levels of enantioselectivity (entries 3, 5, 7, and 8). Employing (*S*,*S*)-DIOP as a chiral catalyst resulted in no desired product (entry 9). Use of the chiral bisamine (DHQ)_2_PHAL provided a lower yield, with no improvement in enantioselectivity (entry 10). Catalysis by the (*R*,*R*)-DACH-napthyl Trost ligand, a chiral bisamidophosphine, resulted in a much inferior yield, with no enantioselectivity (entry 11). Surprisingly, the aminophosphine **42**, which has the same rigid carbon framework and distance between the two functional groups as that in DIOP, catalyzed the reaction successfully, providing a good yield and a slightly improved enantioselectivity (entries 9 and 12). This result offered us hope that we could develop an asymmetric variant of the double-Michael addition using a chiral aminophosphine as the catalyst.

**Table 1:** Preliminary screening with commercially available chiral bisphosphines [29]. 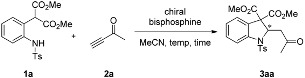

Entry	Catalyst		Temp (°C)	Time (h)	Yield (%) ^a^	ee (%)
1	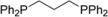	DPPP	80	9	81	0
2	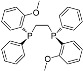	(*S*,*S*)-DIPAMP	80	7	80	0
3			rt ^b^	48	87	5
4	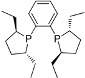	(*R*,*R*)-Et-DuPHOS	80	9	51	0
5			rt	9	58	5
6	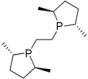	(*S*,*S*)-Me-BPE	80	7	72	0
7			rt	9	78	5
8	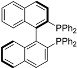	(*R*)-BINAP	rt	9	66	5
9	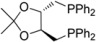	(*S*,*S*)-DIOP	rt	24	n/r ^c^	n/a ^d^
10	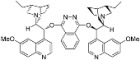	(DHQ)_2_PHAL	rt	9	46	5
11	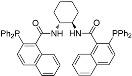	(*R*,*R*)-DACH- napthyl Trost ligand	rt	16	21	0
12 ^e^	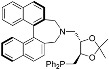	(4*S*,5*R*)-42	rt	24	86	6

^a^ Isolated yield. ^b^ rt = Room temperature. ^c^ n/r = No reaction. ^d^ n/a = Not applicable. ^e^ Synthetically prepared phosphine.

### 2.1. Synthesis of Aminophosphines

**Scheme 2:**
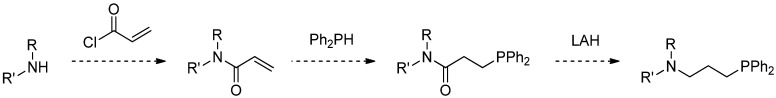
General route toward a 3-amino-1-phosphinopropane.

Scheme 2 outlines our approach for the syntheses of three-carbon-tethered aminophosphines. It starts with acylation of a primary or secondary amine with acryloyl chloride to form the corresponding acrylamide, followed by Michael addition of diphenylphosphine to generate an amidophosphine [30], which would eventually be converted to the aminophosphine through LiAlH_4_-mediated reduction. Because the chirality of the amine would be endowed to the reactive phosphonium center, our goal was to identify potential chiral amines from either commercial or synthetic sources. Notably, we could also test the amidophosphine, an immediate precursor of the aminophosphine, for its enantioinduction ability in double-Michael reactions.

### 2.2. Syntheses of Chiral Aminophosphines from a Commercially Available Chiral Amine

**Scheme 3:**
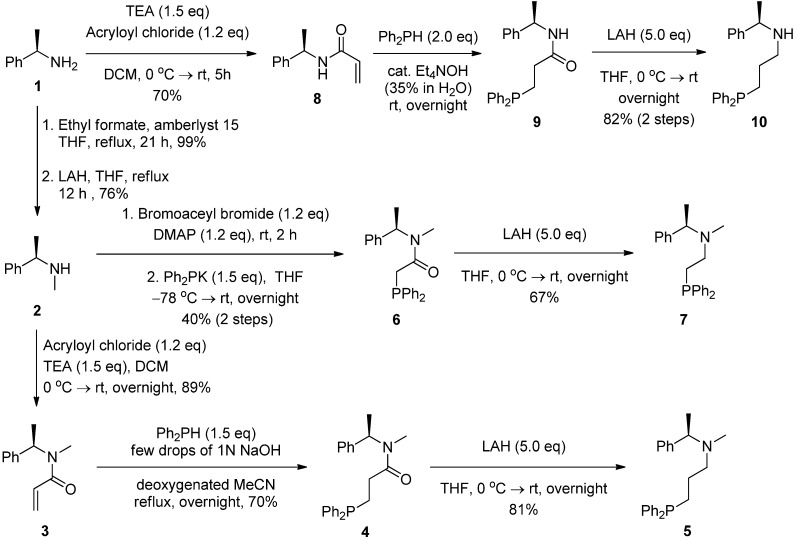
Syntheses of two- and three-carbon-tethered aminophosphines.

To test the viability of the proposed synthetic route, we employed an inexpensive chiral amine **1** to synthesize the chiral aminophosphine **5** (Scheme 3). The chiral amine **1** was first N-methylated via formylation followed by LiAlH_4_-mediated reduction to furnish the chiral amine **2**, which we then treated with acryloyl chloride to prepare the acrylamide substrate **3** for subsequent Michael addition of diphenylphosphine. We obtained our target aminophosphine **5** after LiAlH_4_-mediated reduction of the Michael adduct amidophosphine **4**. This synthetic route was reliable and the purification of the products was straightforward. When testing the chiral aminophosphine **5** in the double-Michael addition, we detected an enantioselectivity of 5% ee. The reaction yield was, however, low, due to our early termination of the reaction to determine the enantioselectivity (Table 2, entry 1).

**Figure 3:**
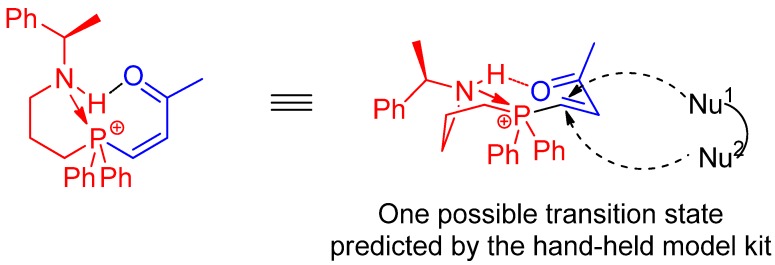
Transition intermediate featuring intramolecular hydrogen bonding.

Shortening the tether length in the aminophosphine would bring the two functional groups in closer proximity, potentially enhancing the interaction between amino and phosphino centers and forming more-rigid reaction intermediates. Accordingly, we synthesized the aminophosphine **7** from the amine **2** through coupling with bromoacetyl bromide, substitution with potassium diphenylphosphide, and LiAlH_4_-mediated reduction of the amidophosphine **6**. To our dismay, when we employed this chiral aminophosphine **7** in the double-Michael reaction, the enantioselectivity of the reaction was barely detectable (Table 2, entry 2). We surmised that it might be more advantageous to feature a free N–H group in the aminophosphine to provide a site for hydrogen bonding to the C=O oxygen atom of the alkynone substrate [31,32,33,34,35]; such hydrogen bonding would bring the two functional groups closer together in the transition intermediate (Figure 3). Thus, we treated the chiral amine **1** with acryloyl chloride to prepare the acrylamide **8**; Michael addition with diphenylphosphine under basic conditions furnished the amidophosphine **9**, which underwent LiAlH_4_-mediated reduction to generate the target chiral aminophosphine **10**. The enantioinduction in the double-Michael reaction catalyzed by the aminophosphine **10** was similarly low to that induced by the aminophosphine catalyst **5**, which features no N–H bond (Table 2, entry 3). Interestingly, the amidophosphines **4**, **6**, and **9** also catalyzed the double-Michael reaction, with yields and enantioselectivities comparable to those of their corresponding aminophosphines (Table 2, cf. entries 1–3 and 14–16).

**Table 2:** Screening with synthetic chiral aminophosphines and amidophosphines. 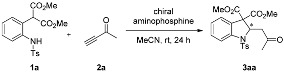

**Entry**	**Aminophosphine**	**Yield (%) a**	**ee (%) b**	**Entry**	**Amidophosphine**	**Yield (%) a**	**ee (%) b**
1 ^b^	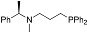	46	5	14	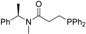	39	5
2	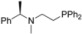	86	0	15	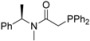	95	0
3	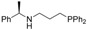	84	4	16	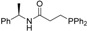	85	4
4	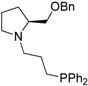	83	3	17	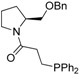	91	0
5	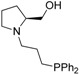	89	6	18	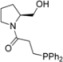	94	9
6	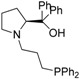	74	2	19	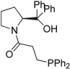	86	0
7	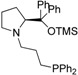	73	2	20	n/a ^c^	n/a ^d^	n/a ^d^
8	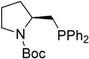	84	-9	21	n/a ^c^	n/a ^d^	n/a ^d^
9	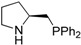	91	-2	22	n/a ^c^	n/a ^d^	n/a ^d^
10	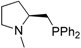	83	0	23	n/a ^c^	n/a ^d^	n/a ^d^
11	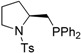	86	-6	24	n/a ^c^	n/a ^d^	n/a ^d^
12	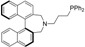	69	10	25	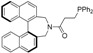	81	0
13	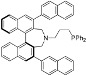	90	3	26	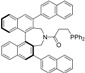	88	3

^a^ Isolated yield. ^b^ Reaction time was 6 h. ^c^ n/a = Not available. ^d^ n/a = Not applicable.

### 2.3. Syntheses of l-Proline-Derived Chiral Aminophosphines

**Scheme 4:**
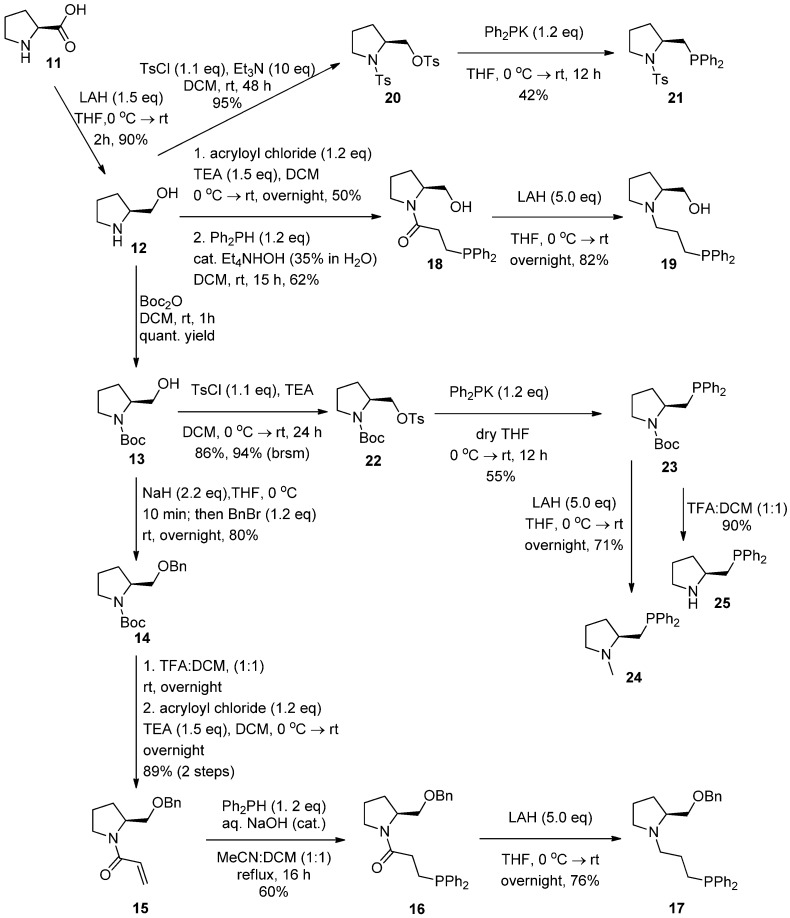
L-Proline-derived chiral aminophosphines.

Because our simple versions of aminophosphines with acyclic chiral amine motifs did not work well as catalysts for the asymmetric double-Michael additions, we decided to insert a more-rigid chiral amine motif derived from L-proline, which has been employed frequently in asymmetric reactions [36,37]. *O*-Benzyl aminophosphine **17 **became our first synthetic target (Scheme 4). After a simple sequence of LiAlH_4_-mediated reduction, N-Boc protection, and O-benzylation, we obtained the globally protected L-prolinol **14**. The Boc group of **14** was released under acidic conditions and the resulting free amine converted to the acrylamide **15** upon treatment with acryloyl chloride. Michael addition of diphenylphosphine to the acrylamide **15** under basic conditions cleanly afforded the amidophosphine **16**, which underwent LiAlH_4_-mediated reduction to yield the target aminophosphine **17**. Unfortunately, we detected only 3% ee when applying the aminophosphine **17** to catalyze the double-Michael reaction. The corresponding amidophosphine **16** provided undetectable enantioselectivity, although the product yield was higher than that obtained with the aminophosphine **17** (Table 2, entries 4 and 17). 

**Figure 4:**
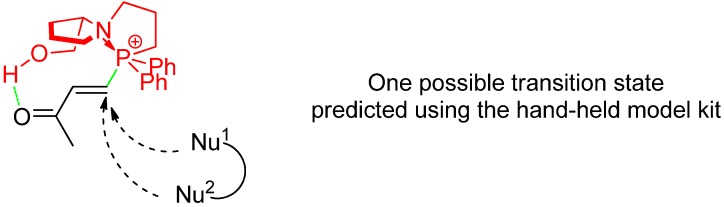
Proposed conformation of an intermediate stabilized through intramolecular hydrogen bonding and intramolecular anchimeric assistance.

We suspected that unmasking the OH group in the aminophosphine **17** might potentially provide hydrogen bonding to the C=O oxygen atom in the alkynone substrate, possibly helping to lock the reaction intermediates into a more-rigid conformation and, hopefully, create a more-asymmetric environment in the subsequent steps toward the final product (Figure 4) [31,32,33,34,35]. The hydroxymethyl aminophosphine **19** was quickly accessible from the unprotected L-prolinol **12** through the sequence of N-acryloylation, Michael addition of diphenylphosphine, and LiAlH_4_-mediated reduction. The resulting enantioselectivities were slightly increased and encouraging: 6% ee for the aminophosphine **19** and 9% ee for the corresponding amidophosphine **18** (Table 2, entries 5 and 18). Notably, the yield and enantioselectivity provided by the amidophosphine **18** were superior to those induced by the aminophosphine **19** in the double-Michael addition.

Another scaffold, based on amino acid-derived phosphines, has recently been employed in asymmetric nucleophilic catalysis [33,34,35]. In this scaffold, the two functional groups are tethered through two carbon atoms, with the chiral element residing on the tether bridge. The aminophosphines **21** and **23**-**25** are derived accordingly from L-proline. The *N*-Tosyl aminophosphine **21** was quickly prepared from L-prolinol **12** after global tosylation and subsequent direct displacement of the tosylate group with potassium diphenylphosphide. The aminophosphines **24** and **25** were both obtained from the aminophosphine **23**, through LiAlH_4_-mediated reduction and acidic Boc-deprotection, respectively; the aminophosphine **23** itself was quickly accessible through tosylation of *N*-Boc-L-prolinol **13** and subsequent substitution with potassium diphenylphosphide. To our dismay, these L-proline-derived aminophosphines provided generally unsatisfactory levels of enantioselectivity in the double-Michael reaction, with the exception of **23** (Table 2, entries 8-11).

**Scheme 5:**
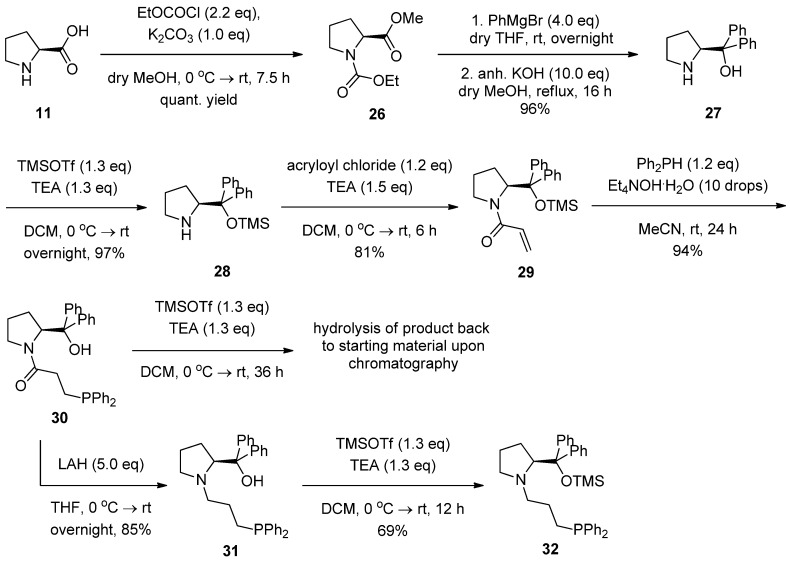
Elaboration of an L-proline-derived aminophosphine.

Introduction of the more substituents to the chiral element in L-proline-derived aminophosphine could possibly enhance the catalyst enantioinduction. Aminophosphine **31** and **32** were thus synthesized as illustrated in Scheme 5. α,α-Diphenyl L-prolinol trimethylsilyl ether **28**, which was easily prepared from L-proline (**11**) using a reported procedure, was subjected to the aforementioned route of phosphine synthesis including acryloylation and Michael-addition of diphenylphosphine. Unexpectedly, TMS protecting group was cleaved under basic Michael-addition condition to yield the hydroxy amidophosphine **30**. The reintroduction of the silyl protecting group to amidophosphine **30** was not achievable due to the hydrolysis of the product during chromatography. Amidophosphine **30** was then set forward to LAH reduction to provide aminophosphine **31** which could be silylated to furnish *O*-TMS aminophosphine **32**. Yet, the application of aminophosphine **31** and **32** to the double-Michael reaction only provided lower reaction yield and enantioselectivity (Table 2, entries 6 and 7). Likewise, amidophosphine **30** resulted in no enantioinduction despite higher yield than the corresponding aminophosphine **31** in the double-Michael reaction (Table 2, entry 19). 

### 2.4. Syntheses of Binol-Derived Chiral Aminophosphines

**Scheme 6:**
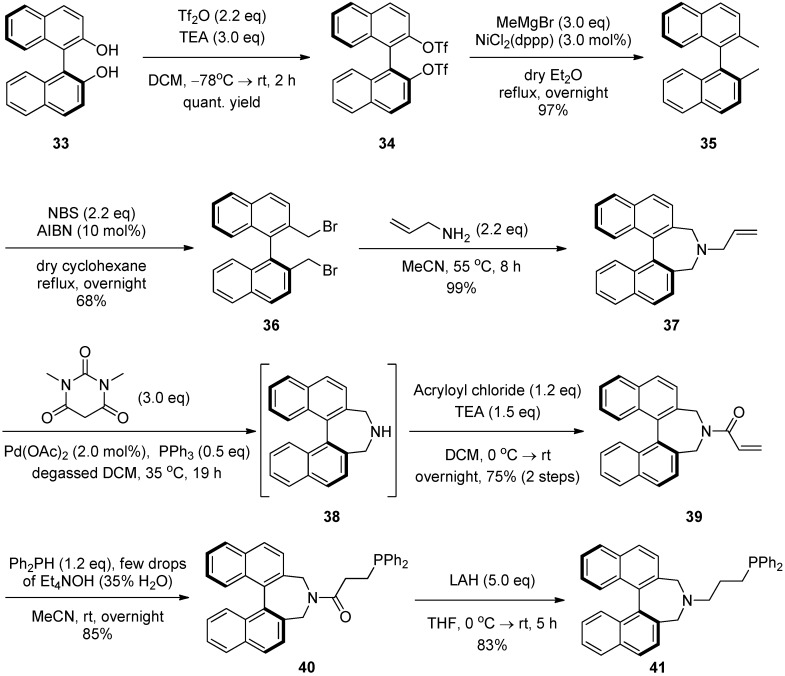
Synthesis of a binol-derived aminophosphine.

Because the L-proline-derived aminophosphines were not satisfactory sources of asymmetric induction in the double-Michael reaction, we sought to prepare binol-derived aminophosphines featuring local *C*_2_ symmetry on the amino moiety. Chiral binol and its derivatives have been used as sources of asymmetry in several chiral catalysts [38,39,40,41]; therefore, we selected binol as our first choice for the preparation of *C*_2_-symmetric amine-containing aminophosphines. Scheme 6 outlines our synthesis of the aminophosphine **41**. Within five steps, using a known procedure, we obtained the local *C*_2_-symmetric amine **38** from commercially available (*S*)-binol (**33**). We subjected the crude product of the amine **38** directly to acryloylation to cleanly yield the acrylamide **39**. Subsequent Michael addition of diphenylphosphine and LiAlH_4_-mediated reduction afforded the amidophosphine **40** and the aminophosphine **41**, respectively. Our earlier version of the binol-derived aminophosphine **42** (Table 1, entry 18) is distinct from aminophosphine **41** in that the latter possesses a shorter, more-flexible tethering carbon atom chain. The aminophosphine **41** provided better enantioselectivity toward the double-Michael reaction, albeit with lower yield, than the aminophosphine **42 **(cf. Table 1, entry 12 with Table 2, entry 12). The amidophosphine **40**, however, provided undetectable enantioselectivity in the double-Michael reaction, even though its reaction yield was higher than that of the aminophosphine **41** (Table 2, entry 25).

**Figure 5:**
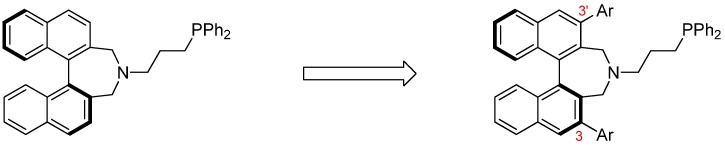
Elaboration of the *C*_2_-symmetric element in the aminophosphine.

Encouraged by a slight improvement in enantioselectivity, we considered modifying the aminophosphine **41** to hopefully further enhance the enantioselectivity of the double-Michael addition. The introduction of an aryl group to binaphthyl systems at the 3 and 3' positions is commonly applied to enhance the steric bias of the molecule and increase the reaction’s enantioselectivity (Figure 5) [42,43]. We decided to install naphthyl groups on the binaphthyl framework because of the ready availability of the requisite reagent and the medium size of the naphthyl group.

**Scheme 7:**
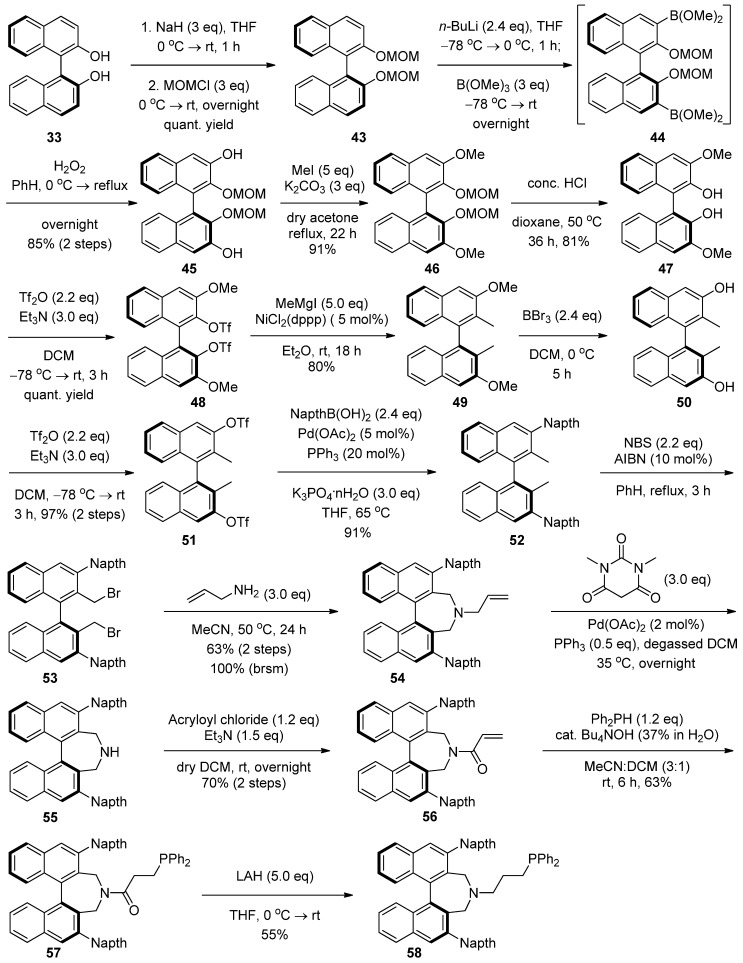
Synthesis of a modified binol-derived aminophosphine.

Using a reported multistep synthesis, we installed naphthyl substituents at the desired 3,3'-positions to obtain the intermediate amine **55**. Subjecting the crude amine **55** to the aforementioned conditions for aminophosphine synthesis, we eventually isolated the amidophosphine **57** and the aminophosphine **58** (Scheme 7). Surprisingly, the aminophosphine **58** provided only 3% *ee*, lower than the enantioselectivity obtained when using the unmodified aminophosphine **41** (Table 2, entries 12 and 13). Similarly, application of the amidophosphine **57** to the double-Michael reaction gave a yield of 88% and an enantioselectivity of only 3% *ee* (Table 2, entry 26).

## 3. Experimental

### 3.1. General

All reactions were performed in flamed-dried round-bottom flasks under an atmosphere of Ar with dry solvents, unless otherwise noted. A glass water condenser, fitted with a rubber septum, was attached to each flask in the cases of reactions performed under reflux. A syringe pump and stainless-steel needles were used for slow addition of reagents into the reaction mixtures. Reactions were monitored through thin-layer chromatography (TLC) on 0.25-mm SiliCycle silica gel plates, visualizing under UV light or staining with iodine, *p*-anisaldehyde, or potassium permanganate. Flash column chromatography (FCC) was performed using SiliCycle Silica-P Flash silica gel (60 Å pore size, 40–63 μm) and compressed air. Purification of phosphines was performed through quick FCC; phosphine-containing fractions were collected immediately into a flask secured under Ar. Organic solvents were evaporated in rotary evaporators under reduced pressure; the flasks were refilled with Ar when isolating phosphines. Phosphine products were stored under Ar at all times.

### 3.2. Materials and Reagents

Reagents were used as received from commercial sources, unless otherwise noted. Acryloyl chloride and potassium phosphate tribasic n-hydrate were purchased from Fluka. Tetraethylammonium hydroxide (37% w/w, aqueous solution), trifluoroacetic anhydride, *N*,*N*-dimethylbarbituric acid, methyl iodide, and (*R*)-(+)-1-phenylethylamine **1** were purchased from Alfa Aesar. Bromoacetyl bromide, lithium aluminum hydride (LAH), potassium diphenylphosphide (0.5 M in THF), methylmagnesium bromide (3.0 M in diethyl ether), hydrogen peroxide (30–32 wt %, solution in water), *n*-butyllithium (1.6 M in hexanes), sodium hydride (60% dispersion in mineral oil), methoxymethyl chloride, methyl magnesium iodide (3.0 M in diethyl ether), azobisisobutyronitrile (AIBN), boron tribromide, allylamine, and L-proline (**11**) were purchased from Aldrich. Trimethyl borate, ethyl formate, and NiCl_2_(dppp) were purchased from Acros Organics. Palladium(II) acetate was purchased from Strem Chemicals. 2-Naphthaleneboronic acid and (*S*)-(–)-1,1'-bi-2-naphthol (**33**) were purchased from Combi-Blocks. Trifluoromethanesulfonic anhydride was purchased from Oakwood. Phenylmagnesium bromide was freshly prepared before use. *N*-Bromosuccinimide was recrystallized from distilled water. TMSOTf was prepared using a published procedure [44,45]. Diphenylphosphine was prepared and purified using published procedures [46,47]. Allylamine was redistilled prior to use. Acetonitrile (MeCN), dichloromethane (DCM), and triethylamine (TEA) were distilled from CaH_2_ under Ar atmosphere. Tetrahydrofuran (THF) and diethyl ether (Et_2_O) were distilled from Na and benzophenone under Ar atmosphere. Dry MeCN and dry DCM were deoxygenated using three freeze/pump/thaw degassing cycles.

### 3.3. Instrumentation

IR spectra were recorded using a Perkin–Elmer Paragon 1000 FTIR spectrometer. NMR spectra were recorded using Bruker Avance-500, ARX-500, or Avance-300 instruments, calibrated to signals from the solvent as an internal reference [7.26 (residual CHC_l3_) and 77.00 (CDC_l3_) ppm for ¹H and ¹³C-NMR spectra, respectively]. Data for ^1^H-NMR spectra are reported in terms of chemical shift (δ, ppm), multiplicity, coupling constant (Hz), and integration. Data for ^13^C-NMR spectra are reported in terms of chemical shift (δ, ppm), multiplicity, and coupling constants (Hz) in the case of *J*_CP_ coupling. The following abbreviations are used to denote multiplicities: s = singlet; d = doublet; t = triplet; q = quartet; qi = quintet; m = multiplet; br = broad; app = apparent. Mass spectra were analyzed using instrument-supplied software. Gas chromatography/mass spectrometry (GC–MS) data were obtained using an Agilent 6890–5975 GC–MS system equipped with an autosampler and an HP5 column; samples were dissolved in DCM.

### 3.4. Procedures for Syntheses of Amidophosphines and Aminophosphines

The amine **2** [48] and the acrylamide **8** [49] in Scheme 3 were prepared following reported procedures. Compounds **12** [50], **13** [51], **14** [52], **20** [53], **21** [54], **22**, **23**, **25** [55], and **24** [56] in Scheme 4 provided spectral data matching those reported in the literature. Compounds **26**, **27** [57], and **28** [58] in Scheme 5 were prepared following reported procedures. Compounds **34**–**38** and **43**–**55** were prepared following published procedures [43].

*(R)-N-Methyl-N-(1-phenylethyl)acrylamide* (**3**). The amine **2** (676 mg, 5 mmol) and dry DCM (20 mL) were placed in a flame-dried flask containing a stirrer bar. Et_3_N (1.5 equiv.) was added to the solution and then the reaction mixture was cooled to 0 °C. Acryloyl chloride (1.2 equiv.) was added and then the mixture was stirred at room temperature overnight under Ar. The reaction was quenched through the addition of aqueous ammonium chloride; the mixture was extracted with DCM (2 × 10 mL) and the organic phases combined, dried (Na_2_SO_4_), and concentrated. The residue was purified through column chromatography (SiO_2_; 30% EtOAc/Hex) to afford a pale yellow oil **3** (842.1 mg, 89%). ^1^H-NMR (300 MHz, CDCl_3_) *δ *(both rotamers) 7.28 (br s, 5H), 6.71–6.52 (m, 1H), 6.39–6.28 (m, 1H), 6.09 (q, *J* = 6.9 Hz, 0.6H), 5.69 (app d, *J* = 8.7 Hz, 1H), 5.23 (q, *J* = 6.0 Hz, 0.4H), 2.69 (s, 1H), 1.58 (d, *J* = 6.0 Hz, 1H), 1.48 (d, *J* = 6.9 Hz, 2H); ^13^C-NMR (75 MHz, CDCl_3_) *δ* (both rotamers) 167.2, 166.4, 140.4, 140.2, 128.7, 128.5, 128.2, 128.1, 127.6, 127.3, 126.5, 54.9, 50.6, 29.6, 28.1, 17.6, 15.5.

*(R)-3-(Diphenylphosphino)-N-methyl-N-(1-phenylethyl)propanamide* (**4**). The acrylamide **3** (568 mg, 3 mmol) and Ph_2_PH (783 μL, 4.5 mmol) were placed in a flask containing deoxygenated MeCN (15 mL). Aqueous 1 N NaOH (13 drops) was added and then the mixture was heated under reflux overnight. After cooling, the reaction mixture was washed with water, dried (Na_2_SO_4_), and concentrated. The residue was purified through column chromatography (SiO_2_; 30% EtOAc/Hex) to afford a colorless oil **4** (788.4 mg, 70%). ^1^H-NMR (500 MHz, CDCl_3_) *δ *(both rotamers) 7.52 (br s, 5H), 7.38 (br s, 9H), 7.3 (br s, 4H), 7.18 (br s, 0.8H), 6.13 (q, *J* = 7.3 Hz, 0.7H), 4.99 (q, *J* = 7.3 Hz, 0.3H), 2.73 (br s, 1H), 2.57 (br s, 3H), 2.53–2.48 (m, 4H), 1.56 (d, *J* = 7.2 Hz, 1H), 1.51 (d, *J* = 7.2 Hz, 3H); ^13^C-NMR (125 MHz, CDCl_3_) *δ* 172.1 (d, *J* = 14.4 Hz), 140.5, 138.2, 138.1, 138.05, 138.00, 132.7 (d, *J* = 6.5 Hz), 132.6 (d, *J* = 6.5 Hz), 128.60, 128.58, 128.4, 128.35, 128.3, 127.2, 127.1, 50.3, 30.2 (d, *J* = 20.5 Hz), 29.2, 23.0 (d, *J* = 10.9 Hz), 15.5 (rotamer) 172.1 (d, *J* = 14.4 Hz), 140.1, 138.3, 138.2, 138.0, 137.9, 132.6, 132.5, 127.3, 126.2, 54.4, 29.6 (d, *J* = 20.5 Hz), 28.0, 23.4 (d, *J* = 10.9 Hz), 17.6 ; ^31^P-NMR (202 MHz, CDCl_3_) *δ* −14.0, (rotamer) −14.2.

*(R)-3-(Diphenylphosphino)-N-methyl-N-(1-phenylethyl)propan-1-amine* (**5**). A solution of the amidophosphine **4** (750.8 mg, 2 mmol) in dry THF (10 mL) was cannulated into a slurry of LAH (379.5 mg, 10 mmol) in dry THF (10 mL) at 0 °C. The mixture was then stirred at room temperature overnight before being cooled at 0 °C and having the reaction quenched through slow addition of 1 N NaOH (5 mL, 5 mmol). The reaction mixture was dried with Na_2_SO_4_ (vigorous stirring for 20 min), then filtered through Celite and washed with Et_2_O (3 × 10 mL). After concentrating the filtrate, the residue was purified through column chromatography (SiO_2_; 3% Et_3_N in 10% EtOAc/Hex) to afford a colorless oil **5** (585.5 mg, 81%). ^1^H-NMR (500 MHz, CDCl_3_) *δ *7.46–7.42 (m, 5H), 7.36–7.32 (m, 10H), 3.57 (q, *J *= 6.7 Hz, 1H), 2.56–2.50 (m, 1H), 2.42–2.37 (m, 1H), 2.17 (s, 3H), 2.10–1.98 (m, 2H), 1.66–1.58 (m, 2H), 1.37 (d, *J* = 6.7 Hz, 3H); ^13^C-NMR (125 MHz, CDCl_3_) *δ *143.9, 138.93 (d, *J* = 13.3 Hz), 138.87 (d,*J* = 13.3 Hz), 132.7 (d, *J* = 4.4 Hz), 132.6 (d, *J* = 4.4 Hz), 128.4, 128.3, 128.2, 128.0, 127.6, 126.6, 63.1, 55.2 (d, *J* = 13.5 Hz), 38.3, 25.5 (d, *J* = 11.0 Hz), 23.5 (d, *J* = 16.0 Hz), 18.3; ^31^P-NMR (202 MHz, CDCl_3_) *δ* −15.1; GCMS (EI+) calcd for [C_9_H_4_Cl_2_O]: *m*/*z *361.2, found 361.3.

*(R)-2-(Diphenylphosphino)-N-methyl-N-(1-phenylethyl)acetamide* (**6**). The amine **2** (676 mg, 5 mmol) was added to a solution of DMAP (733 mg, 6 mmol) in dry THF (50 mL). The mixture was cooled to 0 °C and then bromoacetyl bromide (1.21 g, 6 mmol) in dry THF (20 mL) was added slowly. After stirring at room temperature for 2 h, the mixture was filtered through a short pad of silica gel, which was washed with Et_2_O until the product had completely eluted out. The filtrate was concentrated and then replenished with dry THF (50 mL). The mixture was cooled to –78 °C and then Ph_2_PK (0.5 N in THF, 7.5 mmol, 15 mL) was added slowly. The mixture was stirred overnight at room temperature and then it was quenched (saturated NH_4_Cl), washed (water), dried (Na_2_SO_4_), and concentrated. The residue was purified through column chromatography (SiO_2_; gradient 20–50% EtOAc/Hex) to afford a colorless oil **6** (513 mg, 40%). ^1^H-NMR (300 MHz, CDCl_3_) *δ *(both rotamers) 7.54–7.47 (m, 6H), 7.35–7.19 (m, 15H), 6.03 (q, *J* = 7.1 Hz, 1H), 5.33 (q, *J* = 7.1 Hz, 0.4H), 3.32 (s, 0.8H), 3.23 (s, 2H), 2.67 (s, 1.2H), 2.65 (s, 3H), 1.56 (d, *J* = 7.0 Hz, 1.2H), 1.42 (d, *J* = 7.0 Hz, 3H); ^13^C-NMR (75 MHz, CDCl_3_) *δ* (both rotamers) 169.84 (d, *J* = 7.8 Hz), 169.8 (d, *J* = 7.8 Hz), 140.6, 140.3, 138.2 (d, *J* = 14.3 Hz), 138.0 (d, *J* = 14.3 Hz), 133.0, 132.7, 128.9, 128.7, 128.6, 128.5, 128.4, 127.5, 127.3, 127.2, 126.5, 50.5, 35.7, 35.4, 30.4, 30.3, 28.2, 17.9, 15.5; ^31^P-NMR (121 MHz, CDCl_3_) *δ* −18.8, (rotamer) −18.7.

*(R)-2-(Diphenylphosphino)-N-methyl-N-(1-phenylethyl)ethanamine* (**7**). Using the procedure described for the synthesis of the aminophosphine **5**, the aminophosphine **7** (67%) was obtained as a colorless oil. ^1^H-NMR (300 MHz, CDCl_3_) *δ *(both rotamers) 7.40–7.34 (m, 4H), 7.33–7.19 (m, 11H), 3.56 (q, *J* = 6.8 Hz, 1H), 2.67–2.43 (m, 2H), 2.31–2.16 (m, 5H), 1.30 (d, *J* = 6.8 Hz, 3H); ^13^C-NMR (75 MHz, CDCl_3_) *δ *144.0, 138.7 (d, *J* = 12.6 Hz), 138.6 (d, *J* = 12.6 Hz), 132.8 (d, *J* = 1.9 Hz), 132.5 (d, *J* = 1.9 Hz), 128.5, 128.48, 128.44, 128.35, 128.2, 127.6, 126.8, 63.1, 51.0 (d, *J* = 22.9 Hz), 38.5, 25.9 (d, *J* = 12.0 Hz), 18.9; ^31^P-NMR (121 MHz, CDCl_3_) *δ* −19.9.

*(R)-3-(Diphenylphosphino)-N-(1-phenylethyl)propanamide* (**9**). Using the procedure described for the synthesis of the aminophosphine **10**, but stopping at the first step, the amidophosphine **9** was obtained as a colorless oil. ^1^H-NMR (300 MHz, CDCl_3_) *δ* 7.45–7.39 (m, 4H), 7.36–7.27 (m, 11H), 5.64 (d, *J* = 7.3 Hz, 1H), 5.10 (app qi, *J* = 7.3 Hz, 1H), 2.43–2.36 (m, 2H), 2.29–2.19 (m, 2H), 1.46 (d, *J* = 6.9 Hz, 3H); ^13^C-NMR (75 MHz, CDCl_3_) *δ* 171.2 (d, *J* = 12.8 Hz), 143.1, 137.9 (d, *J* = 12.6 Hz), 132.9, 132.6, 128.7 (d, *J* = 7.1 Hz), 128.5 (d, *J* = 6.6 Hz), 127.4, 126.2, 48.9, 32.9 (d, *J* = 18.3 Hz), 23.4 (d, *J* = 12.1 Hz), 21.7; ^31^P-NMR (121 MHz, CDCl_3_) *δ*−15.4.

*(R)-3-(Diphenylphosphino)-N-(1-phenylethyl)propan-1-amine* (**10**). Ph_2_PH (0.7 mL, 4 mmol) was added to a solution of the acrylamide **8** (350 mg, 2 mmol) in dry MeCN (10 mL). Et_4_NOH**·**H_2_O (37% aqueous solution, 0.5 mL) was added and then the mixture was stirred at room temperature overnight before being concentrated. The residue was dissolved in DCM (20 mL), washed with water (5 mL), dried (Na_2_SO_4_), and concentrated. The residue was dissolved in dry THF (5 mL) and then cannulated into a flask containing a slurry of LAH (380 mg, 10 mmol) in dry THF (10 mL) under Argon. After overnight stirring at room temperature, the reaction mixture was cooled to 0 °C and 1N NaOH (4 mL, 4 mmol) was added to quench the reaction. Na_2_SO_4_ was added and the mixture was stirred vigorously for 20 min, then filtered through Celite and washed with Et_2_O (3 × 10 mL). The filtrate was concentrated under vacuo and the residue was purified through column chromatography (SiO_2_; 5% Et_3_N in 30% EtOAc/Hex) to afford a colorless oil **10** (569.8 mg, 82%). ^1^H-NMR (500 MHz, CDCl_3_) *δ* 7.48–7.44 (m, 4H), 7.39–7.27 (m, 11H), 3.77 (q, *J* = 6.5 Hz, 1H), 2.67–2.55 (m, 2H), 2.15–2.03 (m, 2H), 1.72–1.57 (m, 2H), 1.38 (d, *J* = 6.5 Hz, 3H); ^13^C-NMR (125 MHz, CDCl_3_) *δ *145.6, 138.7 (d, *J* = 12.3 Hz), 138.6 (d, *J* = 12.3 Hz), 132.7 (d, *J* = 4.2 Hz), 132.5 (d, *J* = 4.2 Hz), 128.4, 128.3, 128.2, 126.7, 126.4, 58.0, 48.7 (d, *J* = 13.6 Hz), 26.5 (d, *J* = 16.0 Hz), 25.6 (d, *J* = 11.5 Hz), 24.2; ^31^P-NMR (202 MHz, CDCl_3_) *δ* −14.6.

*(S)-1-(2-((Benzyloxy)methyl)pyrrolidin-1-yl)prop-2-en-1-one* (**15**). TFA (25 mL) was added to a solution of **14** (1.19 g, 6.6 mmol) in DCM (25 mL) and then the mixture was stirred for 5 h at room temperature. The solution was concentrated and the residue poured into a premixed solid comprising NaHCO_3_ and a few pieces of ice; the aqueous phase was extracted with DCM, dried (Na_2_SO_4_), and concentrated. The residue was dissolved in dry DCM (50 mL); TEA (1.4 mL, 10 mmol) was added to the solution, which was then cooled to 0 °C. Acryloyl chloride (0.66 mL, 8.2 mmol) was added and then the mixture was stirred at room temperature overnight before being filtered through a short pad of silica gel and washed with DCM (4×) until the product had eluted completely. The filtrate was concentrated and the residue purified through column chromatography (SiO_2_; 30% EtOAc/Hex) to afford **15** (1.44 g, 89%) as a pale yellow oil. ^1^H-NMR (300 MHz, CDCl_3_) *δ *(both rotamers) 7.37–7.28 (m, 5H), 6.56–6.32 (m, 2H), 5.68–5.59 (m, 1H), 4.53 (s, 0.5H), 4.51 (s, 1.5H), 4.42–4.36 (m, 0.5H), 4.18–4.11 (m, 0.5H), 3.70 (dd, *J* = 9.4, 3.1 Hz, 0.5H), 3.61–3.32 (m, 3.5H), 2.12–1.97 (m, 2H), 1.94–1.84 (m, 2H); ^13^C-NMR (75 MHz, CDCl_3_) *δ* (both rotamers) 164.9, 164.7, 138.6, 137.8, 129.1, 128.8, 128.5, 128.3, 127.8, 127.6, 127.5, 127.4, 73.4, 73.2, 71.7, 70.1, 56.9, 56.8, 47.4, 46.0, 29.0, 27.5, 24.2, 21.9.

*(S)-1-(2-((Benzyloxy)methyl)pyrrolidin-1-yl)-3-(diphenylphosphino)propan-1-one* (**16**). Ph_2_PH (1.2 mL, 6.9 mmol) and 1 M NaOH (10–15 drops) were added to a solution of **15** (1.35 g, 5.5 mmol) in dry MeCN (10 mL) and dry DCM (10 mL). The mixture was stirred at room temperature overnight before being concentrated and purified through column chromatography (SiO_2_; 50% EtOAc/Hex) to afford **16** (1.42 g, 60%) as a colorless oil. ^1^H-NMR (300 MHz, CDCl_3_) *δ *(both rotamers) 7.47–7.40 (m, 4H), 7.36–7.23 (m, 11H), 4.54 (d, *J* = 12.0 Hz, 0.7H), 4.47 (d, *J* = 12.0 Hz, 0.7H), 4.41 (d, *J* = 12.0 Hz, 0.3H), 4.35 (d, *J* = 12.0 Hz, 0.3H), 4.30–4.23 (m, 0.7H), 3.93–3.85 (m, 0.3H), 3.65 (d, *J* = 3.3 Hz, 0.3H), 3.62 (d, *J* = 3.3 Hz, 0.7H), 3.50 (d, *J* = 6.88 Hz, 0.6H), 3.47 (d, *J* = 6.88 Hz, 0.4H), 3.31–3.18 (m, 2H), 2.52–2.27 (m, 4H), 2.05–1.82 (m, 4H); ^13^C-NMR (75 MHz, CDCl_3_) *δ* (both rotamers) 171.1 (d, *J* = 14.5 Hz), 138.6, 138.4 (d, *J* = 16.7), 138.2 (d, *J* = 16.7 Hz), 132.9, 132.9 (d, *J* = 3.8 Hz), 132.7, 132.6 (d, *J* = 3.8 Hz), 128.72, 128.70, 128.53, 128.5, 128.4, 128.3, 127.54, 127.5, 73.3, 73.2, 71.4, 70.1, 56.9, 56.7, 47.2, 47.8, 31.4, 31.2, 28.8, 27.6, 24.1, 23.4, 22.8, 22.7, 21.9; ^31^P-NMR (121 MHz, CDCl_3_) *δ* −15.2, (rotamer) −15.3.

*(S)-2-((Benzyloxy)methyl)-1-(3-(diphenylphosphino)propyl)pyrrolidine* (**17**). Using the procedure described for the synthesis of the aminophosphine **5**, the aminophosphine **17** (76%) was obtained as a colorless oil. ^1^H-NMR (300 MHz, CDCl_3_) *δ *7.44–7.40 (m, 4H), 7.33 (br s, 11H), 4.52 (s, 2H), 3.50–3.46 (m, 1H), 3.37–3.13 (m, 1H), 3.05–3.03 (m, 1H), 2.97–2.91 (m, 1H), 2.64–2.62 (m, 1H), 2.41–2.35 (m, 1H), 2.14–2.09 (m, 2H), 2.05–1.98 (m, 1H), 1.95–1.84 (m, 1H), 1.72–1.60 (m, 5H); ^13^C-NMR (75 MHz, CDCl_3_) *δ *139.1 (d, *J* = 20.1 Hz), 138.9 (d, *J* = 19.9 Hz), 138.6, 132.9 (d, *J* = 15.8 Hz), 132.6 (d, *J* = 15.5 Hz), 128.5, 128.4, 128.3, 127.7, 127.5, 74.0, 73.3, 63.6, 56.8 (d, *J* = 13.8 Hz), 54.4, 28.6, 25.9 (d, *J* = 11.3 Hz), 25.4 (d, *J* = 16.1 Hz), 23.0; ^31^P-NMR (121 MHz, CDCl_3_) *δ* −15.9.

*(S)-3-(Diphenylphosphino)-1-(2-(hydroxymethyl)pyrrolidin-1-yl)propan-1-one* (**18**). Using the procedures described for the syntheses of **3** and **4**, **18** was obtained as a colorless oil. IR (film) *ν*_max_ 3385, 3051, 2951, 2875, 1621, 1434, 1313, 1188, 1052 cm^-1^; ^1^H-NMR (500 MHz, CDCl_3_) *δ *7.46–7.42 (m, 4H), 7.35–7.32 (m, 6H), 4.97 (br s, 1H), 4.19–4.14 (m, 1H), 3.65–3.64 (m, 1H), 3.55–3.51 (m, 1H), 3.37–3.28 (m, 2H), 2.42–2.35 (m, 4H), 2.01–1.95 (m, 1H), 1.91–1.83 (m, 1H), 1.83–1.75 (m, 1H), 1.59–1.52 (m, 1H); ^13^C-NMR (125 MHz, CDCl_3_) *δ *173.4 (d, *J* = 14.3 Hz), 137.8 (d, *J* = 12.1 Hz), 137.8 (d, *J* = 12.1 Hz), 132.7, 132.5, 128.7, 128.4 (d, *J* = 6.5 Hz), 67.2, 61.2, 47.8, 31.3 (d, *J* = 20.3 Hz), 28.1, 24.2, 22.7 (d, *J* = 10.6 Hz); ^31^P-NMR (202 MHz, CDCl_3_) *δ* −14.3.

*(S)-(1-(3-(Diphenylphosphino)propyl)pyrrolidin-2-yl)methanol* (**19**). Using the procedure described for the synthesis of the aminophosphine **5**, the aminophosphine **19** (76%) was obtained as a colorless oil. ^1^H-NMR (500 MHz, CDCl_3_) *δ *7.44–7.39 (m, 4H), 7.32 (br s, 6H), 3.62 (dd, *J* = 10.5, 3.2 Hz, 1H), 3.37 (d, *J* = 10.5 Hz, 1H), 3.05–3.01 (m, 1H), 2.85–2.79 (m, 1H), 2.54–2.51 (m, 1H), 2.36–2.31 (m, 1H), 2.17–2.11 (m, 2H), 2.05–1.99 (m, 1H), 1.88–1.81 (m, 1H), 1.79–1.73 (m, 1H), 1.71–1.66 (m, 2H), 1.64–1.58 (m, 2H); ^13^C-NMR (125 MHz, CDCl_3_) *δ *138.6 (d, *J* = 21.5 Hz), 138.5 (d, *J* = 21.5 Hz), 132.7, 132.6, 132.4, 128.5, 128.4, 128.3 (d, *J* = 6.9 Hz), 128.27 (d, *J* = 6.9 Hz), 64.6, 61.7, 55.0 (d, *J* = 13.1 Hz), 53.8, 27.4, 25.6 (d, *J* = 11.1 Hz), 25.1 (d, *J* = 15.6 Hz), 23.4; ^31^P-NMR (202 MHz, CDCl_3_) *δ* −15.4.

*(S)-1-(2-(Diphenyl((trimethylsilyl)oxy)methyl)pyrrolidin-1-yl)prop-2-en-1-one* (**29**). Using the procedure described for the synthesis of the acrylamide **15**, the acrylamide **29** (81%) was obtained as a colorless oil. ^1^H-NMR (500 MHz, CDCl_3_) *δ *(both rotamers) 7.47–7.25 (m, 10H), 6.81 (dd, *J* = 16.8, 12.3 Hz, 0.7H), 6.29 (dd, *J* = 16.8, 2.0 Hz, 0.7H), 6.25 (dd, *J* = 16.8, 12.3 Hz, 0.3H), 6.12 (dd, *J* = 16.8, 2.0 Hz, 0.3H), 5.63–5.52 (m, 1H), 5.39 (dd, *J* = 12.3, 2.0 Hz, 0.7H), 5.10 (dd, *J* = 12.3, 2.0 Hz, 0.3H), 3.77–3.76 (m, 0.7H), 3.42–3.39 (m, 0.3H), 3.89–3.87 (m, 0.3H), 2.19–2.15 (m, 0.7H), 2.09–2.07 (m, 0.7H), 1.92–1.88 (m, 0.3H), 1.86 (br s, 1H), 1.62–1.58 (m, 0.3H), 1.51–1.46 (m, 0.7H), 1.35–1.29 (m, 0.3H), 1.20–1.14 (m, 0.7H), −0.13 (s, 2.7H), −0.25 (s, 6.3H); ^13^C-NMR (125 MHz, CDCl_3_) *δ* (both rotamers) 166.01, 166.00, 144.3, 144.2, 142.3, 141.0, 130.4, 129.6, 129.3, 129.2, 128.8, 128.1, 127.6, 127.4, 127.3, 127.2, 127.0, 126.4, 125.7, 84.4, 65.9, 62.4, 48.2, 46.3, 28.3, 27.1, 23.8, 21.8, 1.8.

*(S)-3-(Diphenylphosphino)-1-(2-(hydroxydiphenylmethyl)pyrrolidin-1-yl)propan-1-one* (**30**). Using the procedure described for the synthesis of the amidophosphine **18**, the amidophosphine **30** (94%) was obtained as a colorless oil. ^1^H-NMR (500 MHz, CDCl_3_) *δ *7.43–7.25 (m, 20H), 6.92 (s, 1H), 5.16–5.13 (m, 1H), 3.19–3.17 (m, 1H), 2.78–2.77 (m, 1H), 2.34–2.30 (m, 3H), 2.23–2.21 (m, 1H), 2.04–2.02 (m, 1H), 1.93–1.92 (m, 1H), 1.50–1.43 (m, 1H), 0.94–0.91 (m, 1H); ^13^C-NMR (125 MHz, CDCl_3_) *δ* 174.8 (d, *J* = 15.0 Hz), 146.0, 143.3, 137.8 (d, *J* = 12.6 Hz), 132.7 (d, *J* = 11.9 Hz), 132.5 (d, *J* = 11.8 Hz), 128.74, 128.71, 128.5, 128.4, 128.0, 127.8, 127.6, 127.3, 127.14, 127.12, 81.9, 66.8, 48.4, 31.5 (d, *J* = 19.9 Hz), 29.4, 23.1, 22.9 (d, *J* = 11.3 Hz); ^31^P-NMR (202 MHz, CDCl_3_)*δ* −14.3.

*(S)-(1-(3-(Diphenylphosphino)propyl)pyrrolidin-2-yl)diphenylmethanol* (**31**). Using the procedure described for the synthesis of the aminophosphine **19**, the aminophosphine **31** (85%) was obtained as a white crystalline solid.^ 1^H-NMR (500 MHz, CDCl_3_) *δ *7.63 (d, *J* = 7.9 Hz, 2H), 7.56 (d, *J* = 7.9 Hz, 2H), 7.42–7.30 (m, 12H), 7.26 (t, *J* = 7.9 Hz, 2H), 7.21–7.16 (m, 2H), 4.85 (s, 1H), 3.85–3.83 (m, 1H), 3.13–3.10 (m, 1H), 2.34–2.29 (m, 1H), 2.26–2.21 (m, 1H), 2.00–1.87 (m, 2H), 1.82–1.60 (m, 5H), 1.56–1.50 (m, 1H), 1.39–1.31 (m, 2H); ^13^C-NMR (125 MHz, CDCl_3_) *δ* 148.0, 146.4, 138.7 (d, *J* = 16.7 Hz), 138.6 (d, *J* = 16.7 Hz), 132.7, 132.5 (d, *J* = 5.0 Hz), 132.3, 128.4, 128.29, 128.27, 128.22, 128.18, 127.9 (d, *J* = 4.4 Hz), 126.0, 125.6, 125.5, 77.8, 71.0, 57.4 (d, *J* = 14.2 Hz), 55.1, 29.4, 25.2 (d, *J* = 10.7 Hz), 24.9 (d, *J* = 15.6 Hz), 24.4; ^31^P-NMR (202 MHz, CDCl_3_)*δ* −14.6. 

*(S)-2-(Diphenyl((trimethylsilyl)oxy)methyl)-1-(3-(diphenylphosphino)propyl)pyrrolidine* (**32**). TMSOTf (255 μL, 1.4 mmol) was added to a solution of the aminophosphine **31** (335.7 mg, 0.7 mmol) and TEA (0.3 mL, 2.1 mmol) in dry DCM (5 mL) at 0 °C. The mixture was stirred overnight and then concentrated. The residue was purified through column chromatography (SiO_2_; 15% EtOAc/Hex) to afford **32** (266.5 mg, 69%) as a colorless oil. ^1^H-NMR (500 MHz, CDCl_3_) *δ *7.61–7.58 (m, 2H), 7.53–7.50 (m, 2H), 7.46–7.42 (m, 4H), 7.41–7.37 (m, 6H), 7.34–7.31 (m, 3H), 7.29–7.27 (m, 3H), 3.75 (dd, *J* = 9.7, 3.2 Hz, 1H), 2.95 (q, *J* = 9.7 Hz, 1H), 2.66 (dt, *J* = 8.9, 2.2 Hz, 1H), 2.52 (dt, *J* = 11.3, 5.6 Hz, 1H), 2.13–2.08 (m, 2H), 1.94–1.86 (m, 1H), 1.79–1.70 (m, 2H), 1.52–1.44 (m, 2H), 1.36–1.30 (m, 1H), 0.67–0.57 (m, 1H), −0.12 (s, 9H); ^13^C-NMR (125 MHz, CDCl_3_) *δ* 144.4, 143.7, 139.2 (d, *J* = 13.6 Hz), 139.0 (d, *J* = 13.6 Hz), 132.7, 132.6, 132.4, 129.6 (d, *J* = 4.8 Hz), 128.3, 128.2, 128.16, 126.74, 126.71, 84.5, 72.1, 59.0 (d, *J* = 14.5 Hz), 54.5, 28.7, 25.5, 25.5, 25.3, 23.7, 2.0; ^31^P-NMR (202 MHz, CDCl_3_)*δ* −14.4.

*1-(3H-*Dinaphtho[2,1-c:1´,2´-e]*azepin-4(5H)-yl)prop-2-en-1-one* (**39**). Using the procedure described for the synthesis of the acrylamide **29**, the acrylamide **39** (75%) was obtained as a light-yellow foam. ^1^H-NMR (500 MHz, CDCl_3_) *δ *8.00–7.95 (m, 4H), 7.65 (d, *J* = 8.4 Hz, 1H), 7.52–7.44 (m, 5H), 7.30 (q, *J* = 7.0 Hz, 2H), 6.71 (dd, *J* = 16.9, 10.6 Hz, 1H), 6.35 (dd, *J* = 16.9, 1.4 Hz, 1H), 5.77 (dd, *J* = 10.6, 1.4 Hz, 1H), 5.45 (d, *J* = 13.7 Hz, 1H), 4.79 (d, *J* = 12.8 Hz, 1H), 4.01 (d, *J* = 12.8 Hz, 1H), 3.60 (d, *J* = 13.7 Hz, 1H); ^13^C-NMR (125 MHz, CDCl_3_) *δ* 164.9, 135.2, 134.9, 133.3, 133.2, 132.8, 132.0, 131.3, 131.2, 129.2, 128.6, 128.3, 128.2, 127.9, 127.7, 127.4, 127.2, 126.7, 126.1, 126.0, 125.8, 49.7, 46.5. 

*1-(3H-*Dinaphtho[2,1-c:1',2'-e]*azepin-4(5H)-yl)-3-(diphenylphosphino)propan-1-one* (**40**). Using the procedure described for the synthesis of the amidophosphine **30**, the amidophosphine **40** (85%) was obtained as a white solid. ^1^H-NMR (500 MHz, CDCl_3_) *δ *8.00 (t, *J* = 9.2 Hz, 4H), 7.68 (d, *J* = 8.4 Hz, 1H), 7.56–7.52 (m, 7H), 7.50–7.47 (m, 1H), 7.43–7.39 (m, 7H), 7.35–7.31 (m, 2H), 5.53 (d, *J* = 13.5 Hz, 1H), 4.51 (d, *J* = 12.9 Hz, 1H), 3.88 (d, *J* = 12.9 Hz, 1H), 3.56 (d, *J* = 13.5 Hz, 1H), 2.73–2.66 (m, 1H), 2.57–2.47 (m, 3H); ^13^C-NMR (125 MHz, CDCl_3_) *δ* 170.3 (d, *J* = 15.1 Hz), 138.1 (d, *J* = 18.7 Hz), 137.9 (d, *J* = 18.7 Hz), 135.2, 134.8, 133.3, 133.2, 133.0, 132.7 (d, *J* = 6.4 Hz), 132.6 (d, *J* = 6.4 Hz), 132.0, 131.3, 131.2, 129.2 (d, *J* = 6.4 Hz), 128.7 (d, *J* = 3.6 Hz), 128.5 (d, *J* = 6.6 Hz), 128.2 (d, *J* = 4.3 Hz), 127.7, 127.4, 127.2, 126.7, 126.2, 126.0, 125.8, 49.1, 46.3, 30.6 (d, *J* = 19.9 Hz), 23.2 (d, *J* = 11.0 Hz); ^31^P-NMR (202 MHz, CDCl_3_) *δ* −14.1.

*(S)-4-(3-(Diphenylphosphino)propyl)-4*,*5-dihydro-3H-*dinaphtho[2,1-c:1´,2´-e]*azepine* (**41**). Using the procedure described for the synthesis of the aminophosphine **31**, the aminophosphine **41** (83%) was obtained as a white solid. ^1^H-NMR (500 MHz, CDCl_3_) *δ *7.99 (t, *J* = 8.0 Hz, 4H), 7.51 (q, *J* = 8.0 Hz, 10H), 7.39 (t, *J* = 6.2 Hz, 6H), 7.31 (t, *J* = 7.6 Hz, 2H), 3.67 (d, *J* = 12.4 Hz, 2H), 3.19 (d, *J* = 12.4 Hz, 2H), 2.76–2.70 (m, 1H), 2.56–2.50 (m, 1H), 2.18 (t, *J* = 7.7 Hz, 2H), 1.83–1.76 (m, 2H); ^13^C-NMR (125 MHz, CDCl_3_) *δ* 138.7 (d, *J* = 22.4 Hz), 138.6 (d, *J* = 22.4 Hz), 134.8, 133.5, 133.0, 132.7 (d, *J* = 4.5 Hz), 132.6 (d, *J* = 4.5 Hz), 131.3, 128.4, 128.32, 128.27, 128.15, 128.11, 127.6, 127.3, 125.6, 125.2, 56.3 (d, *J* = 13.9 Hz), 55.2, 25.7 (d, *J* = 11.5 Hz), 24.4 (d, *J* = 16.5 Hz); ^31^P-NMR (202 MHz, CDCl_3_)*δ* −14.7.

*1-(2*,*6-Di(naphthalen-2-yl)-3H-*dinaphtho[2,1-c:1',2'-e]azepin*-4(5H)-yl)-3-(diphenylphosphino)propan-1-one* (**57**). Using the procedure described for the synthesis of the amidophosphine **40**, the amidophosphine **57** was obtained as a light-yellow foam (63%). ^1^H-NMR (500 MHz, CDCl_3_) *δ* 8.06 (s, 1H), 8.03–8.01 (m, 4H), 7.98–7.89 (m, 10H), 7.68 (app t, *J* = 7.7 Hz, 2H), 7.60–7.55 (m, 6H), 7.43–7.38 (m, 3H), 7.35–7.31 (m, 6H), 7.28–7.25 (m, 2H), 7.15 (d, *J* = 8.5 Hz, 1H), 5.00 (app t, *J* = 4.6 Hz, 1H), 4.50 (app dd, *J* = 13.9, 5.6 Hz, 1H), 4.17 (app dd, *J* = 14.1, 3.9 Hz, 1H), 183–1.77 (m, 2H), 1.62–1.53 (m, 2H); ^13^C-NMR (125 MHz, CDCl_3_) *δ* 170.4 (d, *J* = 15.3 Hz), 141.3, 140.8, 139.4, 138.4 (d, *J* = 10.0 Hz), 134.8, 133.23, 133.16, 133.1, 132.9, 132.6 (d, *J* = 4.5 Hz), 132.5 (d, *J* = 4.5 Hz), 132.4, 132.3, 131.9, 131.8, 129.7, 129.0, 128.54, 128.52, 128.33, 128.31, 128.25, 127.98, 127.96, 127.88, 127.7, 127.6, 127.5, 127.3, 126.7, 126.6, 126.42, 126.39, 126.2, 126.13, 126.07, 125.9, 125.5, 125.0, 40.2, 31.9 (d, *J* = 18.1 Hz), 22.8 (d, *J* = 12.0 Hz); ^31^P-NMR (202 MHz, CDCl_3_)*δ* −14.2.

*4-(3-(Diphenylphosphino)propyl)-2*,*6-di(naphthalen-2-yl)-4*,*5-dihydro-3H-*dinaphtho[2,1-c:1',2'-e]*azepine* (**58**). Using the procedure described for the synthesis of the aminophosphine **41**, the aminophosphine **58** (55%) was obtained as a light-yellow foam. ^1^H-NMR (500 MHz, CDCl_3_) *δ *8.11 (br s, 2H), 8.08 (s, 2H), 8.01 (d, *J* = 8.1 Hz, 2H), 7.90–7.82 (m, 7H), 7.58 (d, *J* = 8.6 Hz, 2H), 7.56–7.49 (m, 7H), 7.34 (app t, *J* = 7.4 Hz, 2H), 7.31–7.27 (m, 3H), 7.22 (app t, *J* = 7.3 Hz, 5H), 7.14 (app t, *J* = 7.1 Hz, 2H), 3.99 (d, *J* = 12.4 Hz, 2H), 3.15 (d, *J* = 11.8 Hz, 2H), 2.19–2.15 (m, 1H), 2.07–2.01 (m, 1H), 1.67 (br s, 1H); ^13^C-NMR (125 MHz, CDCl_3_) *δ* 140.3, 138.9, 136.2, 133.2, 132.6, 132.5, 132.4, 132.3, 131.9, 130.8, 129.3, 128.6, 128.4, 128.3, 128.1, 128.09, 128.08, 128.0, 127.7, 127.5, 126.2, 125.9, 125.8, 125.7, 55.0 (d, *J* = 13.2 Hz), 50.6, 25.2 (d, *J* = 11.4 Hz), 23.5 (d, *J* = 15.6 Hz); ^31^P-NMR (202 MHz, CDCl_3_)*δ* −14.3.

## 4. Conclusion

Because the bisphosphine-catalyzed double-Michael reaction is an efficient methodology for synthesizing 5- and 6-membered heterocycles, we sought an asymmetric variant of the reaction, employing a chiral bisphosphine catalyst. Several common and commercially available bisphosphines were only marginally successful at catalyzing a double-Michael indoline synthesis. After establishing that the aminophosphine **42** was as efficient as bisphosphine at facilitating the double-Michael reaction, we prepared a series of chiral aminophosphines.

The synthesis of a chiral aminophosphine is simpler than that of a chiral bisphosphine because of the availability of a wide variety of chiral amines found in Nature and the ease of preparing and handling chiral amines. We prepared a series of aminophosphines featuring tethers of two or three carbon atoms, derived from an acyclic chiral amine, L-proline, and (*S*)-binol, and employed them as catalysts in the double-Michael indoline synthesis. Despite the impractical enantioselectivities provided by these aminophosphines, we are encouraged by some slight improvements in enantioinduction relative to those of bisphosphines. Notably, however, we obtained these poor levels of enantioinduction using only one type of substrate under one specific set of conditions; therefore, we should not dismiss the use of chiral aminophosphines in double-Michael indoline syntheses until we have subjected the systems to further investigation. To the best of our knowledge, no literature precedent exists for chiral induction through intramolecular anchimeric assistance. Therefore, it remains a novel and a very challenging task to discover an appropriate aminophosphine for the asymmetric variant of the double-Michael addition and to verify the feasibility of chiral induction through intramolecular anchimeric assistance.
